# Evaluation of a Generic Bortezomib Molecule in Newly Diagnosed Multiple Myeloma Patients

**DOI:** 10.4274/tjh.galenos.2021.2020.0555

**Published:** 2021-08-25

**Authors:** Sinan Mersin, Ayfer Gedük, Özgür Mehtap, Pınar Tarkun, Serkan Ünal, Merve Gökçen Polat, Kemal Aygün, Emel Merve Yenihayat, Hayrunnisa Albayrak, Abdullah Hacıhanefioğlu

**Affiliations:** 1 Kocaeli University Faculty of Medicine, Department of Hematology, Kocaeli, Turkey

**Keywords:** Bortezomib, Multiple myeloma, Equivalent

## Abstract

**Objective:**

Constantly increasing health expenditures lead to the use of generic molecules and generic versions of bortezomib have been used for a long time. The aim of this study is to retrospectively examine the effectiveness, side effects, and reliability of generic bortezomib in newly diagnosed multiple myeloma (MM) patients.

**Materials and Methods:**

The data of 95 patients who received four cycles of bortezomib as first- or second-line therapy in a single center were retrospectively recorded. Treatment responses, side effects, and progression-free survival (PFS) rates were calculated and compared.

**Results:**

Of the 95 patients, 42 used the original and 53 used the generic molecule. Epidemiological data, MM types, genetic risk groups, laboratory values at diagnosis, and bortezomib treatment lines (as a first line or second) were evaluated and there was no statistical difference between the two groups. When the response rates were evaluated according to International Myeloma Working Group criteria, there was no significant difference (p=0.42). Rates of partial response and higher responses were similar (81% vs. 79.2%, p=0.84). PFS rates were 42.8 months with the original and 37.8 months with the generic molecule (p=0.68). Side effects were seen in 44.2% of all patients, and the most common side effects were neuropathy, cytopenia, and infection. These rates were similar in the two groups (p=0.55).

**Conclusion:**

Although this retrospective study is limited in scope, it is the first study comparing the original molecule of bortezomib with a generic version. There were no statistical differences between the two groups in terms of treatment responses, PFS, or side effects. However, large-scale evaluations will help obtain more data on this subject.

## Abstract (tr)

**Amaç:**

Sürekli artan sağlık harcamaları jenerik moleküllerin kullanımına yol açmaktadır ve bortezomibin jenerik molekülleri uzun süredir kullanılmaktadır. Bu çalışmanın amacı, yeni tanı konmuş multipl myelom (MM) hastalarında jenerik bortezomibin etkinliğini, yan etkilerini ve güvenilirliğini retrospektif olarak incelemektir.

**Gereç ve Yöntemler:**

Tek merkezli çalışmamızda birinci veya ikinci basamak tedavide dört kür bortezomib alan 95 hastanın verileri geriye dönük olarak incelendi. Tedavi yanıtları, yan etkiler ve progresyonsuz sağkalım (PFS) oranları hesaplandı ve karşılaştırıldı.

**Bulgular:**

Doksan beş hastanın 42’si orijinal molekülü, 53’ü eşdeğer molekülü kullanmıştır. Epidemiyolojik veriler, MM tipleri, genetik risk grupları, tanı anındaki laboratuvar değerleri ve bortezomib tedavi sıraları (birinci sıra veya ikinci sıra olarak) değerlendirildiğinde iki grup arasında istatistiksel fark olmadığı izlendi. Uluslararası Myelom Çalışma Grubu kriterlerine göre yanıt oranları değerlendirildiğinde anlamlı bir fark bulunmadı (p=0,42). Kısmi yanıt ve daha yüksek yanıt oranları benzerdi (%81’e karşı %79,2, p=0,84). PFS süreleri orijinal molekül için 42,8 ay ve eşdeğer molekül için 37,8 ay olarak hesaplandı (p=0,68). Yan etkiler tüm hastaların %44,2’sinde görüldü ve en sık görülen yan etkiler nöropati, sitopeni ve enfeksiyondu. Bu yan etkilerin görülme oranları iki grupta benzerdi (p=0,55).

**Sonuç:**

Bu retrospektif çalışma, kapsamı sınırlı olmakla birlikte, orijinal bortezomib molekülünü eşdeğeri ile karşılaştıran ilk çalışmadır. Tedavi yanıtları, PFS veya yan etkiler açısından iki grup arasında istatistiksel fark izlenmemiştir. Ancak geniş çaplı değerlendirmeler bu konuda daha fazla veri elde edilmesine yardımcı olacaktır.

## Introduction

Multiple myeloma (MM) is a plasma cell dyscrasia that constitutes 14% of all hematological malignancies and 20% of mortality due to hematological malignancies. It has no cure with current treatment options. In a study examining the global data of 2016, it was shown that the total incidence of MM was 2.1 per 100000 (95% UI: 1.8-2.3) and that 1.5 per 100000 (95% UI: 1.3-1.7) total deaths were associated with myeloma [1]. The incidence has increased over the past 30 years [2,3].

Proteasome is the main extra lysosomal system of cells and inhibition of this system causes cell cycle arrest and apoptosis mainly in neoplastic cells. Proteasome inhibitors are mainly used for myeloma and lymphoma, and bortezomib was the first one to be used. Although patients became refractory to this treatment after a while, it is still used in the first line of myeloma treatment [4,5,6]. In recent years, new proteasome inhibitors such as carfilzomib and ixazomib have been developed and they are used for these relapsed/refractory MM patients with better treatment response rates [7].

In optimal treatment approaches for newly diagnosed patients, besides good efficiency and reliability, the cost balance should also be taken into account. Overall health expenditures for MM continue to increase with the increase in incidence as well as the costs of new treatments [3]. Because of this increase, the development of generic drugs has become necessary and patients have had to use these generic molecules because of the price differences. However, the use of generic molecules initially creates concerns among physicians in terms of effectiveness and side effects. We planned this study in order to eliminate this uncertainty. Bortezomib has more than 70 generic versions in use and 4 of them have been used here in Turkey. In our center, Borcade is used as a generic, and in this study, we compared this molecule with the original one (Velcade) [4,8].

## Materials and Methods

The files of 340 patients diagnosed with MM between 2011 and 2019 in a single center were retrospectively scanned. Ninety-five patients had received only original (Velcade) or only generic (Borcade) bortezomib treatment for at least 4 cycles in the first or second line of therapy with cyclophosphamide and dexamethasone. VCD therapy was selected rather than VTD (bortezomib, thalidomide, and dexamethasone therapy) mainly because of the reimbursement rules of the country. Original molecule users used bortezomib between March 2011 and March 2017 while generic molecule users used it between May 2015 and February 2019. Of the 95 patients included in the study, 42 used the original molecule and 53 used the generic molecule. Patients who received both original and generic molecules, who received more than one generic molecule, who received bortezomib as a third line of treatment, or who received bortezomib after autologous stem cell transplantation or relapse were excluded from the study.

International Myeloma Working Group (IMWG) diagnostic criteria were applied in the diagnosis of patients while International Staging System criteria were used for staging and genetic risk factors were also considered according to IMWG criteria [9]. Routine laboratory analysis, radiological imaging, and positron emission tomography/computed tomography examinations of the patients were performed in our hospital’s central laboratory, radiology department, and nuclear medicine units, respectively. IMWG response criteria were also used in post-treatment response evaluations. Consent was obtained from the ethics committee of our hospital for the analysis of patient files.

After recording the demographic characteristics of our patients, M protein levels at diagnosis, MM types, disease stages, genetic risk groups, extramedullary involvement and lytic lesions in the bones, laboratory values at the time of diagnosis, and the first treatments received before bortezomib-cyclophosphamide-dexamethasone (VCD) therapy, if any, were recorded. After receiving VCD treatment for 4 cycles, response evaluations were performed for all patients. Some of the patients who responded to treatment underwent transplantation immediately, while some underwent autologous stem cell transplant (ASCT) after continuing the same treatment. This was due to ASCT availability at the time of treatment and treatment being continued to maintain the response rates. Patients who could not undergo ASCT were followed. A small number of patients with or without ASCT received maintenance lenalidomide therapy (10 mg/day for 21 days in a 28-day period as the standard in both groups). The recurrence dates of these patients were recorded for both groups and progression-free survival (PFS) times were calculated. Hematological and non-hematological side effects occurring during treatment were rated and recorded according to the latest National Cancer Institute Common Terminology Criteria for Adverse Events (CTCAE) [10].

### Statistical Analysis

SPSS was used for analysis. The Student t-test was used in the analysis of numerical data with normal distribution, the Mann-Whitney U test was used in the analysis of data that did not fit normal distribution and for more than two categorical variables, the chi-square test was used in the analysis of binary categorical variables, and the Kaplan-Meier test was used for estimating survival times. Values of p<0.05 were considered statistically significant.

## Results

Epidemiological data are given in Table 1. There was no statistical difference in the distribution of patients. Associated p-values are also given.

As can be seen in Table 1, the majority of patients in both groups were male and the average age at diagnosis was over 60. The percentage of stage 2 patients in the original molecule group was 50%, and a more homogeneous distribution was observed in the generic molecule group. When the disease stages were categorized according to treatment lines, even though it was statistically insignificant and there were only 11 patients in the generic group for the second treatment line, the high-risk patient group had a slightly higher percentage in the second treatment line in the generic molecule group (p=0.43 and p=0.49). Otherwise, these distributions were similar to those seen in the main groups to which these patients belonged. When evaluated in terms of MM types, the most common myeloma type was immunoglobulin G kappa in both groups. The rate of high-risk patients was 21.4% in the original molecule group and 37.7% in the generic molecule group. When risk group distributions between treatment lines and high-risk patients were evaluated, there were some differences between subgroups. The subgroups of original molecule users in the first line of treatment and generic molecule users in the second line had higher percentages of high risk than the main groups to which they belonged. The numbers of patients in these subgroups were low and the findings were statistically insignificant, but still worth noting when evaluating the results (p=0.34 and p=0.49). It should also be noted that genetic risk assessment could not be performed for the vast majority of patients. In summary, there was no significant statistical difference between groups and subgroups according to treatment lines. Extramedullary involvement, lytic lesion rates, and mean laboratory values at the time of diagnosis were also similar in the groups.

Bortezomib was used in combination with cyclophosphamide and dexamethasone. The bortezomib dosage was 1.3 mg/m^2^ and it was reduced to 1.1 mg/m^2^ for patients experiencing side effects such as neuropathy. Bortezomib was given subcutaneously to all patients. The cyclophosphamide dosage was 500 mg. The dexamethasone dosage was 40 mg for patients below 60 years of age and 20 mg for older patients. The response rates of the patients to the treatments containing bortezomib that they received in the first or second line are given in Table 2.

The patients’ responses were evaluated after 4 cycles of treatment and another evaluation was performed for patients who underwent more than 4 cycles in the pre-transplantation period. Those rates were determined in the same groups according to IMWG criteria for each patient. When the patients’ responses were evaluated, there were some minor differences between the two molecule groups, but these were not statistically significant (p=0.42). When response rates were divided into two groups as above minimal response or below partial response (PR), the results were similar in the two groups (81% vs. 79.2%, p=0.83).

In Turkey, reimbursement rules did not allow the use of bortezomib as a first line of treatment for a period of time. Patients were able to use bortezomib after at least 2 cycles of a combination treatment that did not contain bortezomib, such as vincristine, doxorubicin, and dexamethasone (VAD). After the reimbursement rules were changed, patients were able to use bortezomib treatment in the first line of treatment. For this reason, 27 of the patients received bortezomib-containing regimens as a second line of therapy after 2 cycles of VAD combination therapy. Eleven of 27 patients were in the generic molecule group and 16 of them were in the original molecule group (p=0.63). Treatment results were also analyzed according to these subgroups. The rate of PR and better responses was higher among those using the generic molecule in the group that received bortezomib treatment as the first line of treatment (81% vs. 76.9%) and among those receiving the original molecule in the group that received bortezomib treatment as the second line (87.5% vs. 72.7%). This may be due to the higher percentage of high-risk patients in those subgroups, but again, this finding was not statistically significant (p=0.69 and p=0.33, respectively).

**Figure 1:**
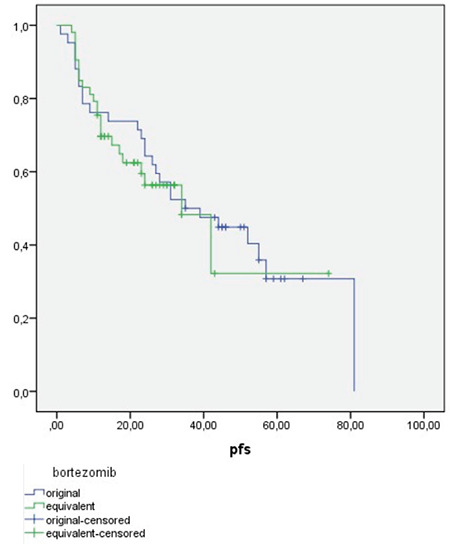
Evaluation of progression-free survival (PFS).

Before the PFS times of the patients were calculated, whether the patients had extra VCD treatment cycles, whether they underwent ASCT, and whether they received maintenance lenalidomide treatment were compared between the groups. Some of the patients in both groups received extra cycles of VCD treatments due to ASCT availability at their time of treatment. The maximum number of treatment cycles was 8 in both groups and means of treatment cycle numbers were 5.2 in the original and 5.7 in the generic molecule group (p=0.89). Twenty-three (54.8%) of the patients using the original molecule and 25 (47.2%) of the patients using the generic molecule underwent transplantation. In both molecule groups, high-dose cyclophosphamide therapies were used for mobilization. All mobilizations succeeded with enough stem cell collection and there was no difference between molecule groups regarding mobilization or transplantation toxicities. After transplantation, all patients in both molecule groups had successful engraftment and no serious complications were seen in either group after transplantation. Twelve (30.8%) of the patients using the original molecule and 13 (24.5%) of the patients using the generic molecule received maintenance lenalidomide treatment. The lenalidomide dosage was 10 mg/day for 21 days in a 28-day period as the standard for all patients. There was no significant difference between the groups in this respect (p=0.537 and p=0.637, respectively). The median follow-up time was longer in the original molecule group, mainly because of treatment dates (30 months vs. 20 months). Considering all these differences, it should be kept in mind that the statistical relations among PFS values are very weak. However, the PFS values were calculated for the two groups to provide a general idea (Figure 1). PFS values were 42.8±4.8 months in the group receiving the original molecule and 38.3±5.89 months in the generic group with no statistically significant difference (p=0.68).

When the side effects related to these molecules were evaluated, side effects were seen in 44.7% of patients using bortezomib. The most common side effects were neuropathy, anemia, thrombocytopenia, neutropenia, and infections. Side effects were observed in 20 patients (47.6%) receiving the original molecule and 22 patients (41.5%) receiving the generic molecule (p=0.55). When side effect grades were evaluated according to the CTCAE, grade 3-4 side effects were observed as neuropathy in 4 patients (2 patients in the original molecule group and 2 patients in the generic molecule group). All remaining observed side effects were grade 1-2. Vincristine therapy (included in the VAD regimen) can also cause permanent neuropathy, so to better separate this from the side effects of bortezomib, neuropathy was evaluated in both first-line and second-line subgroups. In the first-line treatment subgroup, the neuropathy risk was higher in the original molecule group, while the opposite finding was seen for the second-line group. However, these differences were statistically insignificant (23.1% vs. 7.1%, p=0.06 and 18.8% vs. 27.3%, p=0.6, respectively). The side effects seen in both groups and the associated p-values in comparing the groups are given in Table 3.

## Discussion

It is known that the treatment results of patients are improved with new drugs. However, the increase in health expenditures related to these drugs leads health authorities to seek some alternatives. One of the prime examples of this is generic imatinib therapy. It was reported that the responses of chronic myeloid leukemia patients treated with original imatinib were maintained with generic products and annual cost was reduced by 96% [11]. In two real-life studies, generic imatinib was reported to be effective and safe in first-line treatment [11,12]. There are also studies about the economics of generic drugs on a nationwide scale and studies addressing the reasons for choosing generic products [13,14]. Other than imatinib, there are only a few studies comparing generic molecules with their original counterparts. Those studies include comparisons of low-molecular-weight heparin and psychoactive drugs with their generic counterparts [15,16]. As far as we know, our study is the first one comparing original bortezomib with its generic molecule.

Bortezomib was licensed by the Food and Drug Administration in 2003 for patients with MM and it has been used in combination therapy with cyclophosphamide and dexamethasone since 2007 [17,18,19]. In Turkey, the original molecule has been used in combination therapy since 2010. Generic bortezomib was launched with the obtaining of a license in 2012. In accordance with the reimbursement policy of the relevant health authority, it started to be used for patients due to the price difference. However, generic bortezomib caused some concerns among physicians at first regarding its effectiveness and side effect profile.

### Study Limitations

In our study, when the data of these patients were examined, the distributions of epidemiological features, stages, disease types, and other risk factors between groups were not statistically different. When the response rates to treatment were evaluated, there was no statistically significant difference between the two groups in either first-line or second-line treatments. There were some statistically insignificant differences observed between subgroups that may be due to the percentage of high-risk patients. Treatment response rates in both groups were similar to those reported in studies in which the original bortezomib molecule was combined with cyclophosphamide and dexamethasone. In those studies, the rate of any response (PR and above) was 80%, and similar data were obtained in both groups in our study [17,19,20,21]. The results showed us that the responses of patients using the generic bortezomib molecule were similar to those of patients using the original molecule (81% with the original molecule, 79.2% with the generic). Even though these findings were statistically weak, there was also no significant difference between PFS times, and these values were similar to those of other studies on the VCD protocol [22,23]. When side effects were evaluated, no significant difference was found between the two groups. The most common side effects were neuropathy, cytopenia, and infections, similar to other studies, and in our study the side effect of diarrhea was observed less often than in other bortezomib studies [17,19,20,22,23,24].

## Conclusion

MM is a disease in which response rates are increased with new treatments and total survival is prolonged, but there is still no cure. It is a fact that such diseases add additional costs to the health expenditures of countries. Generic medicines can be an alternative both to provide access to new medicines and to reduce the burden on health expenditures. Although this retrospective study includes a limited number of patients, it is the first study of generic bortezomib since it became available for use in Turkey. Our data show that the responses to this drug are similar to those of the original molecule, while the side effects are also similar to those of the original molecule and manageable. Randomized, prospective studies with greater numbers of patients and longer follow-up times are needed to understand whether the use of generic bortezomib in MM treatment affects long-term survival.

## Figures and Tables

**Table 1:**
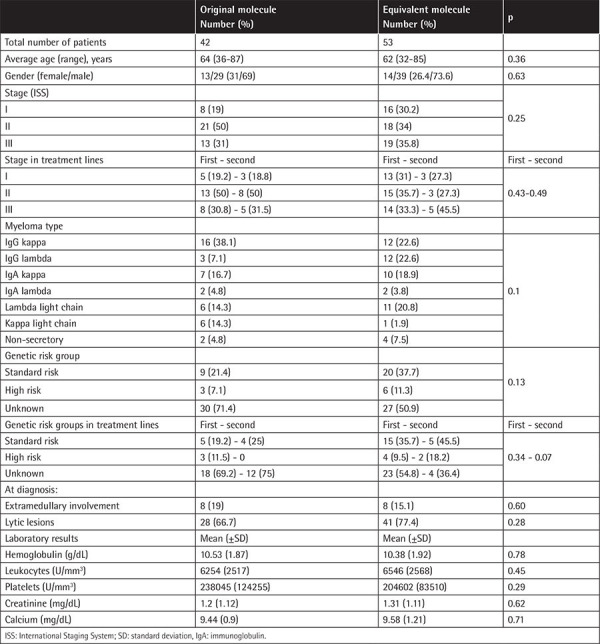
Characteristics of patients.

**Table 2:**
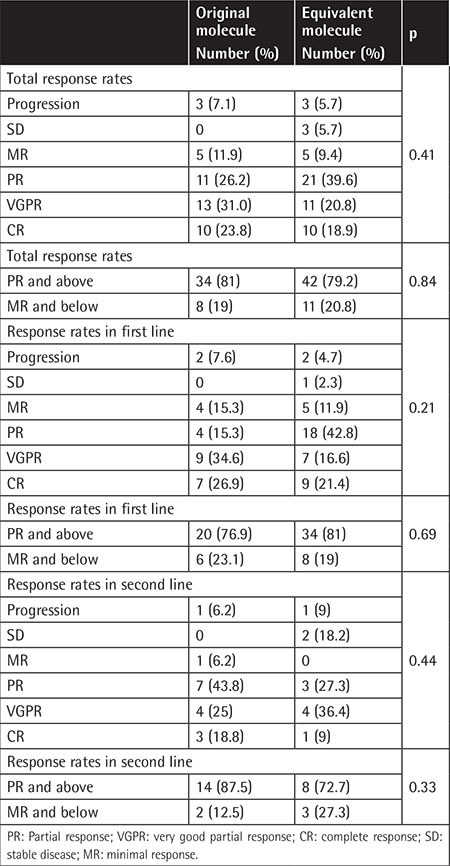
Response to treatments.

**Table 3:**
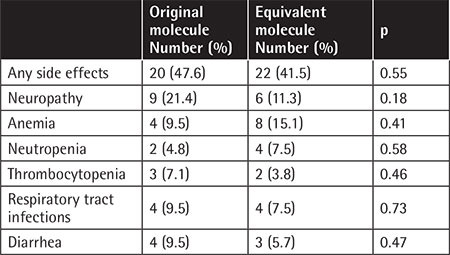
Side effects.
